# Postoperative Delirium and Cognitive Dysfunction After Cardiac Surgery: The Role of Inflammation and Clinical Risk Factors

**DOI:** 10.3390/diagnostics15070844

**Published:** 2025-03-26

**Authors:** Raluca-Elisabeta Staicu, Corina Vernic, Sebastian Ciurescu, Ana Lascu, Oana-Maria Aburel, Petru Deutsch, Elena Cecilia Rosca

**Affiliations:** 1 Doctoral School Medicine-Pharmacy, “Victor Babes” University of Medicine and Pharmacy Timisoara, Eftimie Murgu Square No. 2, 300041 Timisoara, Romania; raluca.staicu@umft.ro (R.-E.S.); sebastian.ciurescu@umft.ro (S.C.); 2 Institute for Cardiovascular Diseases of Timisoara, Clinic of Anesthesia and Intensive Care, “Victor Babes” University of Medicine and Pharmacy Timisoara, Gheorghe Adam Street, No. 13A, 300310 Timisoara, Romania; deutsch.petru@umft.ro; 3 Department III Functional Sciences, Discipline of Medical Informatics and Biostatistics, “Victor Babes” University of Medicine and Pharmacy of Timisoara, Eftimie Murgu Square No. 2, 300041 Timisoara, Romania; 4 Department III Functional Sciences—Pathophysiology, “Victor Babes” University of Medicine and Pharmacy of Timisoara, Eftimie Murgu Square No. 2, 300041 Timisoara, Romania; lascu.ana@umft.ro (A.L.); oanaduicu@umft.ro (O.-M.A.); 5 Centre for Translational Research and Systems Medicine, “Victor Babes” University of Medicine and Pharmacy Timisoara, Eftimie Murgu Square No. 2, 300041 Timisoara, Romania; 6 Institute for Cardiovascular Diseases of Timisoara, “Victor Babes” University of Medicine and Pharmacy Timisoara, Gheorghe Adam Street, No. 13A, 300310 Timisoara, Romania; 7 Advanced Research Center of the Institute for Cardiovascular Diseases, “Victor Babes” University of Medicine and Pharmacy of Timișoara, Eftimie Murgu Square No. 2, 300041 Timisoara, Romania; 8 Department of Surgery X, “Victor Babes” University of Medicine and Pharmacy Timisoara, Eftimie Murgu Square No. 2, 300041 Timisoara, Romania; 9 Department of Neurology, “Victor Babes” University of Medicine and Pharmacy Timisoara, 300041 Timisoara, Romania; rosca.elena@umft.ro; 10 Clinical Emergency County Hospital Timisoara, 300736 Timisoara, Romania

**Keywords:** inflammation, neuroinflammation, cardiac surgery, IL-6, IL17-A, CRP, NLR, SII, SIRI, postoperative cognitive dysfunction, postoperative delirium

## Abstract

**Background/Objectives**: Postoperative delirium (POD) and postoperative cognitive dysfunction (POCD) are prevalent neurological complications following cardiac surgery, significantly affecting patient recovery and long-term outcomes, including increased risk of persistent cognitive impairment, functional decline, and mortality. Understanding the underlying mechanisms and risk factors for POD/POCD is crucial for improving perioperative management. This study aimed to investigate the relationship between postoperative systemic inflammation, assessed through inflammatory markers, and the occurrence of POD and POCD in patients undergoing cardiac surgery. **Methods**: We prospectively enrolled 88 patients aged 18–79 years undergoing open-heart surgery. Patients with preoperative cognitive impairment or high surgical risk (based on EuroSCORE and SOFA scores) were excluded to focus on the impact of inflammation in a relatively unselected cohort. Postoperative inflammatory responses (CRP, NLR, IL-6, IL-17A, SII, and SIRI) were measured, and patients were assessed for POD (CAM-ICU) and POCD (neuropsychological testing) during hospitalization and at 3 months follow-up. Statistical comparisons were performed between patients who developed POD/POCD and those who did not. **Results**: Postoperative inflammation was confirmed across the cohort, with significant increases in CRP, NLR, IL-6, SII, and SIRI. While correlational analyses between changes in individual inflammatory markers and POD/POCD were not statistically significant in the entire cohort, patients who developed POD/POCD exhibited significantly higher levels of IL-6 and NLR at 48 h postoperatively (*p* < 0.05). Established clinical risk factors significantly associated with POD/POCD included older age, prolonged cardiopulmonary bypass (CPB) duration, extended mechanical ventilation, vasopressor support duration, blood transfusion, renal dysfunction, and elevated postoperative creatine kinase (CK) and lactate dehydrogenase (LDH) (*p* < 0.05). Ejection fraction (EF) < 45% and atrial fibrillation (AF) were also more prevalent in the POD/POCD group. **Conclusions**: Our findings emphasize the significant role of the postoperative inflammatory response, particularly IL-6 and NLR, in conjunction with established clinical risk factors, in the development of POD and POCD after cardiac surgery. Postoperative IL-6 and NLR levels, readily measurable and cost-effective markers, may contribute to identifying patients at higher risk. Comprehensive perioperative management strategies targeting inflammation, modifiable clinical risk factors, and organ function are crucial for mitigating POD and POCD and improving cognitive outcomes in this vulnerable population.

## 1. Introduction

Postoperative delirium (POD) and postoperative cognitive dysfunction (POCD) are significant neurological complications following surgery, particularly cardiac surgery, impacting patient outcomes and quality of life. These complications are not only distressing for patients and their families but also pose a substantial burden on healthcare systems due to increased length of hospital stay and higher healthcare costs. Delirium and POCD are highly prevalent in intensive care settings, with reported incidence rates reaching up to 79% for cognitive disorders and 81% for delirium [1].

Delirium is defined in the Diagnostic and Statistical Manual of Mental Disorders (DSM) as a disturbance in attention (reduced ability to direct, focus, sustain, and shift attention) and awareness (reduced orientation to the environment) [2], accompanied by disorganized thinking and further categorized into three subtypes: hyperactive, hypoactive, and mixed. POCD is characterized by a sustained decline in cognitive function manifesting in days, weeks, or months after surgery [3], potentially affecting attention, memory, language, and concentration in the absence of pre-existing neurological pathology.

Several factors contribute to the development of postoperative neurological complications following cardiac surgery, including the complexity of surgical procedures, advanced patient age, pre-existing comorbidities, cardiopulmonary bypass (CPB), and intraoperative hypothermia [4]. Prolonged administration of sedative, hypnotic, and analgesic medications may also play a role. Importantly, patients who develop POD or POCD exhibit a significantly higher 5-year mortality rate [5]. This increased mortality risk, coupled with the detrimental impact on quality of life and autonomy, highlights the critical need for identifying patients at high risk and developing rapid, specific methods for early detection and intervention.

Cardiac surgery carries a notably higher incidence of delirium and POCD compared to other surgical fields, including general, orthopedic, and urological surgeries, even when these involve similarly aged patient populations. Studies report delirium rates of 8.4% to 23.8% after general surgery [6,7], rising to 28% with general anesthesia [7], while orthopedic surgery shows rates around 8.2% [8] and urological surgery between 8.8% and 29%, depending on the intervention type [9]. Overall, in older adults, delirium incidence ranges from 9% to 30% for general surgery, 16–44% for hip fractures, and 11–55% for cardiac surgery [10].

While cardiopulmonary bypass (CPB) is often correlated with this elevated risk in cardiac surgery, the precise role of CPB remains debated. Some studies suggest CPB does not directly impact inflammatory marker levels [11] or POD incidence [12], and off-pump cardiac surgery has not consistently reduced POD rates [13]. However, even when considering other intraoperative factors like hemodynamic instability, surgical bleeding, and anesthesia type, patients can still develop POD and POCD.

Given evidence that inflammatory markers can compromise blood-brain barrier permeability, leading to cerebral edema and neurological dysfunction [4], this study prioritizes the investigation of systemic inflammation as a critical factor contributing to POD and POCD in cardiac surgery. To investigate the role of inflammation and neuroinflammation in POD and POCD after cardiac surgery, we assessed a panel of biomarkers. We focused on composite inflammatory indices, the Systemic Immune-Inflammation Index (SII) and the Systemic Inflammatory Response Index (SIRI), which have shown promise as predictors of postoperative delirium, including in cardiac surgery [14,15,16,17]. These indices are advantageous as they integrate multiple components of the systemic inflammatory response, offering a more comprehensive and robust measure than individual markers. Specifically, SII is calculated from complete blood counts using the formula: SII = (neutrophil count × platelet count)/lymphocyte count [18]. SIRI is similarly calculated as: SIRI = (neutrophil count × monocyte count)/lymphocyte count. To capture the dynamic inflammatory response, we also assessed the percentage change in SIRI from baseline to 48 h postoperatively, calculated as (SIRI 48 h − SIRI baseline)/SIRI baseline) × 100 [14]. The neutrophil-to-lymphocyte ratio (NLR) was also included, as it is associated with POD risk and reflects systemic inflammation [19,20,21].

Furthermore, we measured C-reactive protein (CRP) and interleukin-6 (IL-6), well-established markers of systemic inflammation known to be elevated in POD and dementia [11]. Blocking peripheral IL-6 has even been shown to reduce blood-brain barrier disruption [22], further supporting its relevance. Finally, we analyzed interleukin-17A (IL-17A), a cytokine implicated in the central nervous system (CNS) inflammation, blood-brain barrier (BBB) impairment, and activation of astrocytes and microglia [23,24], suggesting its potential role in neuroinflammation contributing to POD/POCD.

Therefore, this prospective observational study aimed to investigate the correlations between pre- and postoperative levels of systemic inflammatory markers (SII, SIRI, NLR, CRP, IL-6, and IL-17A) and the occurrence of POD and POCD in patients undergoing elective cardiac surgery (patients devoid of any neurological issues preoperatively, without elevated inflammatory markers, who underwent identical anesthesia and cardioplegia and received the same sedation, weaning, and analgesia protocols postoperatively).

## 2. Materials and Methods

### 2.1. Study Design and Setting

We conducted a prospective, observational cohort study at a single center, the Institute of Cardiovascular Diseases in Timisoara, specifically within the Division of Cardio-vascular Anesthesia and Intensive Care Medicine. The study period spanned from 15 February 2024 to 1 September 2024 and included 88 patients undergoing elective cardiac open-heart surgery. Ethical approval was obtained from the Ethics Committee of the Institute of Cardiovascular Diseases from Timisoara (Protocol code 2098/16 March 2022) and the Ethics Committee of Victor Babes University of Medicine and Pharmacy Timisoara (Protocol code 91/29 April 2022). All participants provided written informed consent prior to enrollment.

### 2.2. Participants and Inclusion/Exclusion Criteria

Participants were adult patients aged 18 years or older scheduled for elective cardiac open-heart surgery. We applied specific exclusion criteria to minimize confounding variables and ensure a homogenous study population. Patients were excluded if they presented with any of the following:

- Pre-existing Neurological and Psychiatric Conditions: History of neurodegenerative diseases (e.g., Parkinson’s disease or Alzheimer’s disease), cerebrovascular disease (e.g., stroke or transient ischemic attack), pre-existing diagnosis of dementia or cognitive impairment, or major psychiatric disorders (e.g., schizophrenia, bipolar disorder, or major depressive disorder) requiring ongoing psychotropic medication.
- Active Systemic Illness: Active sepsis, acute or chronic inflammatory diseases requiring immunosuppressive therapy (e.g., rheumatoid arthritis or inflammatory bowel disease), end-stage renal disease requiring dialysis, Child-Pugh Class C liver cirrhosis, or pre-existing hematological malignancies or severe coagulopathies. Furthermore, patients who exhibited elevated inflammatory marker values before surgery were not included in the study.
- Sensory and Substance Use Disorders: Severe vision or hearing impairment that would preclude accurate cognitive assessment (vision impairment uncorrectable to 20/200 in the better eye or hearing impairment requiring hearing aids and still unable to understand conversational speech) or history of chronic alcohol abuse or dependence.
- Perioperative Physiological Instability: Intraoperative mean arterial pressure (MAP) variations greater than 20% of baseline or MAP < 50 mmHg during cardiopulmonary bypass (CPB), preoperative or intraoperative hematocrit <20%, intraoperative hypothermia below 32 °C, or postoperative benzodiazepine administration.
- Pre-existing Cognitive Impairment (Based on Screening): Preoperative cognitive impairment as indicated by a score below 23 points on the Mini-Mental State Exam (MMSE) or below 4 points on the Mini-Cog test. This stricter Mini-Cog cutoff (scores of 3 or less usually indicate dementia) was chosen to conservatively exclude even mild pre-existing cognitive deficits that could confound POCD assessment [25].

### 2.3. Surgical Procedures and Anesthesia

All patients underwent elective open-heart surgery, including coronary artery bypass graft surgery (CABG) and classic and thoracoscopic valve replacements and repairs. Under predefined institutional guidelines for cardiac surgery, general anesthesia was administered using sevoflurane. Typical induction agents included propofol and fentanyl, muscle relaxation was achieved with rocuronium, and anesthesia was maintained with sevoflurane in oxygen and air. Intraoperative analgesia was typically provided with fentanyl or remifentanil. While the specific choice of hypnotic agents, opioids, vasopressors, inotropes, and vasodilators was at the discretion of the attending anesthesiologist, guided by institutional guidelines and individual patient factors, anesthetic management was guided by predefined institutional guidelines. Standard intraoperative monitoring included electrocardiography, pulse oximetry, capnograph, invasive arterial blood pressure monitoring, central venous pressure monitoring, and depth of anesthesia monitoring using Bispectral Index (BIS), and maintaining a target range between 40–60 throughout the anesthetic. During surgery before CPB, a mean arterial pressure (MAP) > 65 mmHg was maintained; during CPB, the target MAP was 50–60 mmHg. Mild hypothermia, targeting a core temperature between 32 °C and 34 °C, was maintained intraoperatively. St. Thomas Hospital cardioplegia solution was administered at 15- to 20-min intervals, using a 1:1 blood/crystalloid ratio for the initial dose and a 4:1 blood/crystalloid ratio for maintenance. The priming solution consisted of 500 mL of Gelofusine, 500 mL of Ringer’s lactate, 20 mg of furosemide, 100 mg of heparin, 1000 mg of cefazolin, and 100 mL of 20% mannitol. A standard roller pump and heat exchanger system were used for cardiopulmonary bypass and cardioplegia delivery.

### 2.4. Postoperative Care and Biomarker Measurements

Postoperatively, patients were transferred to the intensive care unit (ICU) and sedated with propofol via continuous intravenous infusion until they met standard clinical criteria for weaning from mechanical ventilation, as determined by the ICU intensivist. Weaning from mechanical ventilation was conducted according to the intensivist’s clinical judgment based on standard clinical criteria, which typically included adequate oxygenation, stable hemodynamics, and spontaneous breathing efforts. Morphine was administered intravenously as needed for analgesia, as per ICU protocol. Blood samples were collected preoperatively and at 24 and 48 h postoperatively. The SII, SIRI, NLR, and CRP were calculated from complete blood counts performed on these samples. Plasma samples for interleukin measurements were collected in ethylenediaminetetraacetic acid (EDTA)-containing tubes, centrifuged immediately after collection, and stored in vacutainers at −80 °C until analysis, as previously described [26,27]. Interleukin-6 (IL-6) and Interleukin-17A (IL-17A) levels were quantified using commercially available enzyme-linked immunosorbent assay (ELISA) kits: Legend Max Human IL-6 ELISA kit (BioLegend, San Diego, CA, USA, Catalog Number: 430504) and Legend Max Human IL-17A ELISA kit (BioLegend, Catalog Number: 432504).

### 2.5. Postoperative Delirium and Postoperative Cognitive Dysfunction (POD/POCD) Assessment

Patients were monitored postoperatively for the development of both POD and POCD. Delirium assessments were performed twice daily (morning and evening) by trained anesthesia residents or attending anesthesiologists as part of their routine clinical evaluations during ICU rounds. The Richmond Agitation-Sedation Scale (RASS) was used daily to assess sedation level. Patients with RASS scores of −4 or −5 (comatose and unassessable) were not evaluated for delirium using CAM-ICU. For patients with RASS scores of −3 or higher, delirium was assessed using the Confusion Assessment Method for the Intensive Care Unit (CAM-ICU). CAM-ICU assessments were performed twice daily, starting on the day of extubation and continuing for up to 7 days after or until ICU discharge, whichever came first, following an internal standardized protocol based on recommendations from previous studies [28,29]. Changes in CAM-ICU and RASS scores were recorded daily. Motoric subtypes of delirium were defined based on CAM-ICU and RASS scores: hyperactive delirium was defined as CAM-ICU positive with RASS scores between +1 and +4 on each assessment, characterized by restlessness, agitation, and device removal attempts; hypoactive delirium was defined as CAM-ICU positive with RASS scores between 0 and −3 on each assessment, characterized by lethargy, somnolence, and inattention.

Postoperative cognitive dysfunction (POCD) was assessed using a cognitive screening approach employing the MMSE and the Montreal Cognitive Assessment (MoCA). These assessments were administered by trained research personnel blinded to patient biomarker levels and delirium status to minimize bias. The MMSE and MoCA were administered at 96 h postoperatively (for early POCD assessment), at hospital discharge, and again at 3 months. Postoperative cognitive dysfunction (POCD) was diagnosed at 96 h, discharge, and 3 months if patients achieved a score of 24 or less on the MMSE or a score of 26 or less on the MoCA. These cutoff scores are consistent with established thresholds for indicating cognitive impairment in older adults and postoperative populations [30,31]. The MMSE and MoCA are well-established and widely used tools for evaluating global cognitive functioning, covering various cognitive domains relevant to POCD, including memory, attention, language, visuospatial skills, and executive function. While not a comprehensive neuropsychological battery, the combination of MMSE and MoCA provides a sensitive and clinically practical approach for detecting POCD in the postoperative setting. POCD assessments were conducted at hospital discharge and again at 3 months postoperatively. A significant correlation has been observed between the Mini-Mental State Examination (MMSE), the Montreal Cognitive Assessment (MoCA) scores, and educational level, highlighting their impact on test outcomes. The findings in various studies suggest a strong association between educational attainment and the accuracy of diagnoses based on MMSE and MoCA scores [32], and other studies revealed that educational level had a lesser impact on performance in the short memory-recall task compared to age. Moreover, the results suggest that educational attainment plays a more significant role in assessing memory, particularly when memory recall is impaired [33].

In contrast, age appears to have a more pronounced effect on tasks evaluating memory absence [33]. Compared to the MMSE, the MoCA is more effective in detecting mild cognitive impairment (MCI) among younger and highly educated older adults. However, the MMSE demonstrates an advantage over the MoCA in screening for MCI in individuals with lower education levels and older age groups [34].

Cognitive function was assessed using the Romanian translation of the MoCA Version 7.1, obtained from the MoCA website (www.mocatest.org) accessed on 14 March 2025. This version represents a standardized and readily available Romanian translation of the MoCA. It is the officially recommended Romanian version and is widely used in clinical and research settings in Romania. Cognitive abilities were also assessed using the MMSE. While the MMSE is widely used in clinical practice and research in Romania as a commonly employed cognitive screening tool, we recognize that, to our knowledge, a formally validated Romanian version of the MMSE is not currently available.

EuroSCORE II and Sequential Organ Failure Assessment (SOFA) scores were calculated preoperatively and postoperatively to characterize patient risk profiles and overall disease. Nevertheless, patients with a low probability of mortality, as determined by these scores, were included in the study cohort.

### 2.6. Statistical Analysis

Statistical analyses were performed using Microsoft Excel (version 2021) for data management and preprocessing, while JASP (version 0.19.2) was utilized for advanced statistical computations. Continuous variables were summarized using means, standard deviations, and minimum and maximum values, while categorical variables were reported as absolute and relative frequencies. For comparisons between the POD/POCD group and the No POD/POCD group, continuous variables were compared using independent samples *t*-tests when normally distributed or the Mann–Whitney U test for non-normally distributed data. Categorical variables were analyzed using chi-square tests. To examine the relationships between systemic inflammatory markers and postoperative complications, Pearson’s correlation coefficients were computed. These correlations were assessed to determine potential associations between inflammatory markers, cardiovascular parameters, and clinical outcomes. Additionally, multiple logistic regression models were applied to evaluate potential predictors of postoperative outcomes. For between-group comparisons, independent *t*-tests and analysis of variance (ANOVA) were used to assess statistical differences in inflammatory marker levels across patient subgroups. Chi-square tests were employed for categorical variable comparisons. The predictive performance of logistic regression models was evaluated through pseudo-R^2^ metrics, classification accuracy, and area under the curve (AUC) analyses. All statistical tests were conducted at a 95% confidence level, with statistical significance set at *p* ≤ 0.05. Sensitivity analyses were performed to ensure the robustness of findings, considering potential confounders and interactions among variables. Effect sizes were computed using Cohen’s d for continuous variables, with values interpreted as small (0.2), moderate (0.5), or large (0.8) effects. Odds ratios (OR) with 95% confidence intervals were calculated for categorical variables to assess the strength of association between predictor variables and outcomes. We also used multiple comparison correction methods for inflammatory markers.

## 3. Results

The study enrolled 110 patients scheduled for open-heart, CABG, and valve surgeries. After excluding 6 patients lost to follow-up and 16 patients based on preoperative cognitive screening (MMSE < 23 or Mini-Cog < 4), the final analysis included 88 patients aged from 18 to 79 years. Within this cohort of 88 patients, 22 (25%) developed postoperative delirium (POD) or postoperative cognitive dysfunction (POCD) during their hospital stay, and 9 (40.9%) of those assessed at 3 months exhibited cognitive impairment.

### 3.1. Patient Demographics and Clinical Characteristics

Table 1 summarizes the demographic and clinical characteristics of the entire study cohort (*N* = 88).

### 3.2. Postoperative Inflammatory Marker Changes in the Entire Cohort

Analysis of inflammatory markers revealed postoperative changes across the entire cohort. Specifically, we observed a marked increase in CRP, reaching a mean peak of 186.61 mg/dL at 48 h. The NLR peaked at 24 h postoperatively (mean 18.88) before decreasing to 10.29. The SII and SIRI showed substantial increases post-surgery (SII: preoperative mean 465.34, postoperative mean 2326.97; SIRI: preoperative mean 1.38, postoperative mean 15.80). The IL-6 also rose from a preoperative mean of 15.80 pg/mL to a 167.88 pg/mL peak. The IL-17A increased from 4.09 pg/mL to 10.29 pg/mL.

**Figure 1:**
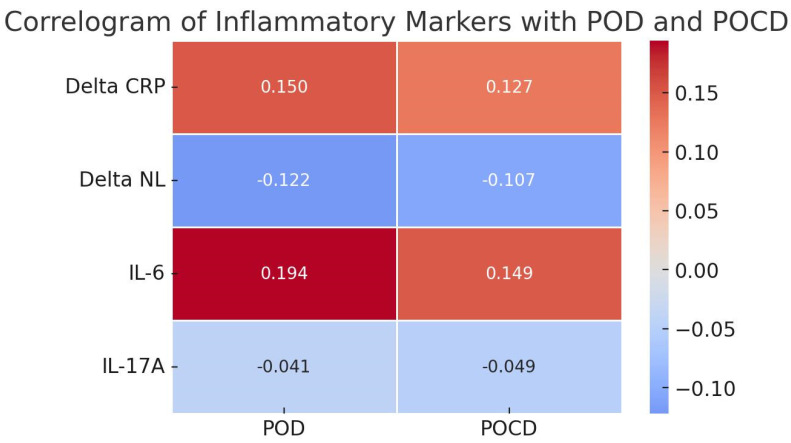
Correlogram of inflammatory markers with POD and POCD. Whole group of patients (*n* = 88).

Figure 1 presents that none of the tested inflammatory markers demonstrated statistically significant correlations with POD or POCD (all *p* > 0.05). Notably, the correlation between Delta IL-6 and POD was weakly positive (r = 0.194) and approached statistical significance (*p* = 0.071), suggesting a potential trend. However, no definitive conclusions can be drawn from this sample regarding the relationship between inflammatory markers and postoperative neurological complications.

The correlation between POD occurrence and Delta PCR is weak and statistically insignificant, indicating that changes in PCR (Delta PCR) over time have no clear association with POD. Regarding POCD, the relationship is direct but weaker than that between POD and Delta PCR, suggesting minimal influence or no direct connection. We observed a weak, negative, and statistically insignificant correlation for NLR, indicating that variations in NLR (Delta NLR) do not significantly impact POD. In relation to POCD, the correlation is negative (inverse) and weak and lacks statistical significance, further suggesting no clear association between NLR changes and POCD. The relationship between POD and IL-6 is direct, yet it approaches statistical significance, indicating a potential link that may become evident in a larger sample size. Conversely, the very weak negative correlation between IL-17A and POD/POCD suggests no meaningful association, implying that IL-17A does not influence these postoperative neurological complications in our study group.

### 3.3. Comparison of Clinical, Surgical, and Biomarker Variables Between Patients With and Without POD/POCD

To identify factors associated with POD/POCD, we compared patients who developed these complications (Group 1: POD/POCD Group, *n* = 22) to those who did not (Group 2: No POD/POCD Group, *n* = 66). Table 2 presents a detailed comparison of demographic, clinical, surgical, and biomarker variables between these two groups, Table 3 summarizes the logistic regression model assessing the presence of POD or POCD. The significant improvement in model fit (*p* < 0.001), along with high pseudo-R^2^ values (McFadden, Nagelkerke, Tjur = 1.000; Cox & Snell = 0.679), indicates strong explanatory power. Table 4 presents the regression coefficients for the variables included in M_1_, highlighting their individual contribution to the model.

Demographic Factors: Patients in the POD/POCD group were significantly older (mean age 66 vs. 60 years, *p* = 0.004, Cohen’s d = −0.726, medium effect). Gender distribution did not differ significantly between groups (*p* = 0.523).

Surgical and Intraoperative Factors: Patients who developed POD/POCD experienced significantly longer cardiopulmonary bypass (CPB) times (mean CPB time 135.3 vs. 107.65 min, *p* < 0.001, Cohen’s d = −0.916, large effect) and required vasopressor support for a longer duration (mean vasopressor support duration 26.61 vs. 17.06 h, *p* < 0.001, Cohen’s d = −1.019, large effect).

Postoperative Factors: The POD/POCD group had significantly longer mechanical ventilation duration (mean MV duration 27 vs. 14.89 h, *p* = 0.004, Cohen’s d = −0.726, medium effect).

Inflammatory Markers and Lab Values: Patients with POD/POCD showed significantly elevated CRP levels at 48 h (mean CRP 217.28 vs. 177.31 mg/L, *p* = 0.020, Cohen’s d = −0.581, medium effect) and higher NLR levels at 24 h (mean NLR 21.4 vs. 15.52, *p* = 0.038, Cohen’s d = −0.519, medium effect) and at 48 h (mean NLR 14.98 vs. 8.81, *p* = 0.013, Cohen’s d = −0.623, medium effect) and higher IL-6 levels at 48 h (mean IL-6 196.94 vs. 160.79 pg/mL, *p* = 0.013, Cohen’s d = −0.623, medium effect). Postoperative creatine kinase (CK) (*p* = 0.023, Cohen’s d = −0.570, medium effect) and lactate dehydrogenase (LDH) levels (*p* < 0.001, Cohen’s d = −0.981, large effect) were also significantly higher in the POD/POCD group.

Renal Function and Blood Transfusion: Both preoperative (*p* < 0.001, Cohen’s d = −0.868, large effect) and postoperative creatinine levels (*p* < 0.001, Cohen’s d = −0.933, large effect) were significantly elevated in the POD/POCD group. A significantly greater proportion of POD/POCD group patients required blood transfusions (81% vs. 45.45%, *p* < 0.001, OR = 2.028).

Cardiac and Other Factors: Atrial fibrillation occurred significantly more frequently in the POD/POCD group (18.18% vs. 15.15%, *p* = 0.001, OR = 1.618). While ejection fraction was lower in the POD/POCD group, this difference approached but did not reach statistical significance (*p* = 0.072).

Non-Significant Variables: In contrast, patient weight, diabetes mellitus prevalence, smoking status, preoperative and 24-h CRP levels, preoperative and postoperative IL-17A levels, preoperative and postoperative SII and SIRI, Delta SIRI, preoperative IL-6 levels, 24-h NLR, and 24-h glucose levels did not differ significantly between the groups (all *p* > 0.05).

### 3.4. Neuropsychological Evaluation

The neuropsychological evaluations, using both the MMSE and MoCA for POCD assessment and CAM-ICU for delirium, revealed the temporal evolution of postoperative neurological complications. The POD, assessed twice daily from extubation using CAM-ICU, was diagnosed in 14 patients (15.9% of the cohort, *N* = 88) at 48 h postoperatively.

Cognitive function was assessed using MMSE and MoCA at 96 h postoperatively to evaluate for early POCD. At 96 h postoperatively, 10 of the 14 patients with POD at 48 h continued to exhibit cognitive dysfunction based on meeting POCD criteria on MMSE and/or MoCA (score ≤ 24 on MMSE or ≤26 on MoCA). Furthermore, by 96 h, an additional 8 patients were newly diagnosed with POCD based on meeting these same POCD criteria on either the MMSE or MoCA. This resulted in a cumulative prevalence of POCD in 22 patients (25% of 88) by 96 h postoperatively.

Overall, 22 patients (25% of 88) experienced either POD or POCD during their postoperative hospitalization up to 96 h. All 22 patients diagnosed with POCD at 96 h were invited and completed the 3-month follow-up assessment. At this later assessment, 9 (40.9%) patients continued to meet the criteria for POCD, while 13 patients no longer met the criteria based on MMSE and/or MoCA scores.

## 4. Discussion

The POD and POCD are significant complications following cardiac surgery, impacting patient recovery and long-term quality of life. This prospective observational study aimed to investigate the relationship between systemic inflammation, as measured by key inflammatory markers, and the occurrence of POD and POCD in patients undergoing open-heart surgery, assessing cognitive function in the immediate postoperative period and at 3 months. Our analysis confirmed a substantial postoperative inflammatory response characterized by marked elevations in markers such as CRP, IL-6, and NLR. While our correlational analysis did not reveal statistically significant associations between individual inflammatory markers and POD/POCD across the entire cohort, our findings suggest that postoperative inflammation, particularly IL-6, may contribute to these neurological complications’ development.

While correlational analyses across the entire cohort did not show statistically significant associations between individual inflammatory markers and POD/POCD, we observed a more significant increase in postoperative IL-6 levels at 48 h in patients who developed POD or POCD compared to those who did not (Table 2). Although the correlation between Delta IL-6 and POD approached but did not reach statistical significance (*p* = 0.071), the significantly elevated IL-6 levels in the POD/POCD group suggest a potential role for IL-6 as a marker of increased risk. Interleukin-6 is a key pro-inflammatory cytokine known to cross the BBB and contribute to neuroinflammation, making it a biologically plausible candidate in the pathogenesis of POD and POCD [35]. The dynamic postoperative increase in IL-6, almost 10-fold compared to preoperative values, further highlights its responsiveness to surgical stress and potential utility as a marker of inflammatory burden. Our findings, combined with existing literature, suggest that postoperative IL-6 levels, particularly in conjunction with established clinical risk factors such as advanced age (over 65 years), reduced ejection fraction (EF < 45%), respiratory dysfunction, prolonged CPB duration, extended mechanical ventilation (over 24 h), and significant blood transfusion, may contribute to a refined risk assessment for POD and POCD in cardiac surgery patients.

In our study, CRP exhibited a marked postoperative increase across the entire cohort, indicative of a robust systemic inflammatory response to cardiac surgery. While correlational analysis across all patients revealed a weak, non-significant correlation between changes in CRP and POD (Figure 1), comparison between groups showed significantly elevated CRP levels at 48 h in patients who developed POD or POCD (Group 1) compared to those who did not (Group 2) (Table 2). This suggests that while CRP, as a general marker of inflammation, rises in all patients after surgery, higher levels at 48 h may be associated with an increased risk of POD/POCD. Our findings are consistent with some literature suggesting that postoperative changes in CRP alone may not be a strong predictor of POD/POCD in cardiac surgery [11], particularly compared to other markers like IL-6. However, CRP’s role might be more pronounced in other contexts, such as when preoperative CRP is elevated, reflecting pre-existing inflammatory burden and potentially increasing vulnerability to postoperative complications [25], or in non-cardiac surgery, where the nature and drivers of inflammation may differ [36].

The NLR also provided insights into postoperative inflammation. Significantly elevated NLR levels were observed at both 24 and 48 h in the POD/POCD group (Group 1) compared to Group 2 (Table 2), further supporting the role of inflammation in postoperative cognitive dysfunction. NLR is a readily available marker reflecting systemic inflammation and the balance between pro-inflammatory neutrophils and anti-inflammatory lymphocytes. Our findings align with a growing body of literature demonstrating the association of increased NLR with POD in non-cardiac [37,38] and cardiac surgery and its potential to predict mortality in cardiothoracic surgery [39]. However, a limitation of our study is the exclusion of patients with preoperative NLR values exceeding ≥ 3.4. This exclusion criterion, implemented to minimize confounding from pre-existing inflammatory conditions, may have limited our ability to capture the spectrum of NLR’s influence fully. By excluding patients with higher baseline inflammation, we might have missed a potentially more vulnerable subgroup where NLR’s predictive value for POD/POCD is even more substantial. Future studies could explore the role of NLR across a broader range of preoperative values, including patients with elevated baseline NLR, to better define its clinical utility. The link between elevated NLR and both cognitive complications and mortality suggests that it may reflect a broader state of systemic vulnerability and heightened surgical risk.

In contrast to IL-6, CRP, and NLR, SII and Systemic SIRI did not show significant differences between groups or significant correlations with POD or POCD in our study. We did observe a strong positive correlation between postoperative SII and SIRI levels (r = 0.81), which is unsurprising given that both are composite indices reflecting related aspects of systemic inflammation. The weak correlation between preoperative SII and SIRI (r = 0.35) and between preoperative SIRI and postoperative mechanical ventilation hours (r = 0.25) suggests that baseline inflammatory status, as captured by these markers, might have a limited direct influence on postoperative ventilation duration in our cohort, with multiple intraoperative factors likely playing a more dominant role in ventilation needs. Interestingly, our study contrasts with findings in the literature [36], which highlight the predictive value of elevated preoperative SII and SIRI for POD risk after cardiac surgery. While we did not find postoperative SII or SIRI significantly associated with POD/POCD outcomes in our cohort, the established literature points toward the importance of preoperative inflammatory status. This discrepancy may suggest that baseline, pre-existing inflammation, captured preoperatively by SII and SIRI, could be a more critical determinant of POD risk than postoperative changes in these markers or that our study lacked the statistical power to detect subtle differences in postoperative SII/SIRI related to POD/POCD. Further research should explore the relative predictive value of preoperative versus postoperative SII and SIRI in larger cohorts and consider preoperative SII/SIRI as potential risk stratification tools.

The strong positive correlation we observed between POD at 48 h and early cognitive dysfunction at 96 h (MMSE and MoCA-based POCD) underscores the close temporal relationship between delirium and early postoperative cognitive impairment in our cardiac surgery cohort. This finding aligns with recent studies demonstrating that immediate postoperative cognitive deficits, including delirium, can have long-lasting consequences for cognitive trajectories, potentially extending up to 5 years postoperatively [40,41]. This emphasizes the clinical importance of early detection and intervention for both POD and POCD to potentially mitigate long-term cognitive decline.

The association between patients who received corticosteroid therapy and those who developed POD at 96 h is r = 0.65, *p* ≤ 0.05 in our study, indicating that corticosteroid therapy alone may not be sufficient to prevent POD, or its effectiveness remains uncertain. This finding aligns with existing literature [38,42,43]. Additionally, other studies suggest that high-dose corticosteroid therapy in cardiac surgery, particularly dexamethasone at a dose of 0.2 mg/kg, may influence the occurrence of early postoperative POCD [40].

Our study demonstrated significantly longer durations of both mechanical ventilation and vasopressor support in patients who developed POD/POCD (Table 2). Patients in the POD/POCD group required an average of 27 h of mechanical ventilation compared to 14.89 h in the No POD/POCD group (*p* = 0.004, Cohen’s d = −0.726) and vasopressor support for 26.61 h versus 17.06 h (*p* < 0.001, Cohen’s d = −1.019). These findings are consistent with established literature linking prolonged mechanical ventilation and vasopressor use [41,44] to increased POD and POCD risk. Mechanistically, prolonged mechanical ventilation can contribute to POD/POCD through multiple pathways, including lung-brain axis inflammation, increased sedation exposure, and prolonged immobility. Similarly, extended vasopressor support, particularly with norepinephrine, may impact cerebral function by disrupting blood-brain barrier integrity in the context of systemic inflammation [45], altering cerebral blood flow, and potentially impairing cerebral autoregulation [46].

Blood transfusion was significantly more frequent in the POD/POCD group. In our study, 81% of patients in the POD/POCD group received blood transfusions, compared to 45.45% in the No POD/POCD group (*p* < 0.001, OR = 2.028). This finding supports the accumulating evidence linking perioperative blood transfusions to an elevated risk of POD and POCD [46]. The causal mechanisms are complex and not fully understood, but transfusion-related immunomodulation (TRIM) and the introduction of inflammatory mediators contained within transfused blood products are proposed mechanisms contributing to neuroinflammation and cognitive dysfunction [47]. Furthermore, several studies have indicated that perioperative blood transfusions are associated with an elevated risk of these complications, particularly when the transfusion volume exceeds 3 units [46]. Other studies have suggested that the risk is amplified after the transfusion of 6 or more packed red blood cell units within 24 h postoperatively [44]. We observed correlations between elevated postoperative LDH levels and the occurrence of POD and POCD at 48 and 96 h. Patients in the POD/POCD group also exhibited significantly higher postoperative LDH levels (Table 2, *p* < 0.001, Cohen’s d = −0.981). Elevated LDH is a marker of cellular damage and tissue hypoxia, suggesting that increased cellular stress and tissue injury associated with cardiac surgery, particularly in those developing POD/POCD, may contribute to systemic stress and potentially neuroinflammation [48].

Our study revealed significantly higher preoperative and postoperative creatinine levels in the POD/POCD group (Table 2) with *p* < 0.001 and Cohen’s d = −0.868, respectively, *p* < 0.001 and Cohen’s d = −0.933. This finding aligns with the known association between acute kidney injury (AKI) and POD [49]. Renal dysfunction can contribute to POD/POCD through multiple mechanisms, including accumulating uremic toxins, electrolyte imbalances, and exacerbating systemic inflammation, all of which can impact brain function [50].

While diabetes mellitus prevalence did not differ significantly between groups in our study, it is important to consider the potential modifying effect of age. Although postoperative glucose control appeared similar between groups, suboptimal preoperative glycemic control, which we did not directly assess, could still be a relevant risk factor. As highlighted in previous research [51], the impact of diabetes on POCD risk may be more pronounced in older individuals, and our cohort had a mean age of 66 years. Future research should explore the role of preoperative glycemic control and potential interactions between diabetes, age, and inflammatory markers in predicting POD/POCD in cardiac surgery.

Clinical implications: the present study highlights the potential clinical relevance of postoperative IL-6 levels as a marker associated with an increased risk of POD and POCD in cardiac surgery. While further research is needed to confirm causality and predictive value, these findings suggest that IL-6 measurement could be integrated into risk stratification protocols to identify high-risk patients who may benefit from enhanced monitoring and preventive strategies. Furthermore, the observed associations of prolonged mechanical ventilation, vasopressor support duration, blood transfusion, and renal dysfunction with POD/POCD underscore the importance of perioperative modifiable factors that can be targeted to reduce cognitive risk.

Our finding that approximately 40% of patients who experienced POCD at 96 h continued to exhibit cognitive dysfunction at 3 months highlights the clinically significant potential for persistent cognitive impairment in a substantial proportion of patients following cardiac surgery who experience early cognitive dysfunction. While the small sample size of the persistent POCD group (*n* = 9) limits statistical power for further analyses of this subgroup, this prevalence estimate provides valuable descriptive data regarding the longer-term burden of cognitive dysfunction in patients with early postoperative neurological complications.

Future Directions: Future research should prioritize larger, multi-center studies to validate our findings and refine the predictive models incorporating IL-6, NLR, and clinical risk factors. Specifically, future research directions include:

- Validation of IL-6 and NLR predictive accuracy: Prospective, multi-center studies to rigorously evaluate the predictive accuracy of postoperative IL-6 levels and NLR, both alone and in combination with clinical risk scores, for POD and POCD development.
- Randomized controlled trials to explore the impact of intervention strategies to minimize modifiable risk factors, such as mechanical ventilation duration, optimize hemodynamic management to reduce vasopressor requirements, and refine transfusion protocols to reduce blood product exposure on POD/POCD incidence.
- Long-term follow-up studies to characterize the cognitive trajectories of patients identified as high risk based on postoperative inflammatory markers and clinical factors and to assess the impact of interventions on long-term cognitive outcomes.
- Further mechanistic studies to elucidate the specific roles of IL-6, NLR, and other inflammatory pathways in the neuroinflammation underlying POD and POCD following cardiac surgery, potentially using preclinical models and advanced biomarker analyses.
- Studies expanding the investigation to include patients with a broader range of preoperative inflammatory marker values, particularly NLR, to fully define their predictive utility and determine if baseline inflammation is a key modifier of postoperative risk. The interpretation of our findings should consider several limitations. As a single-center study with a relatively modest sample size (*N* = 88), our results may not be fully generalizable to broader cardiac surgery populations. The exclusion of patients with elevated preoperative NLR values (≥3.4) to minimize confounding might have limited the spectrum of inflammatory risk captured in our cohort and potentially underestimated the full predictive value of NLR. We used cognitive screening tools (MMSE and MoCA) for POCD assessment rather than a more comprehensive neuropsychological battery, which may have limited the sensitivity to detect subtle cognitive deficits. Furthermore, while we identified associations between inflammatory markers and POD/POCD, our observational design cannot establish causality. Finally, we did not directly assess preoperative glycemic control, which could be a relevant confounding factor, particularly given the known interaction between diabetes, age, and cognitive risk.

Another limitation of our study is using the MMSE without a formally validated Romanian version. While the MMSE is widely used in Romanian clinical practice, the absence of local validation means that its psychometric properties in the Romanian population, including its sensitivity and specificity for detecting cognitive impairment and delirium and its susceptibility to floor effects and biases related to age and education within the Romanian context, are not fully established. Therefore, interpretations of absolute MMSE scores should be made with caution. However, we believe the MMSE still provides valuable information, mainly when focusing on changes in scores over time and group comparisons rather than precise cutoffs and when considered in conjunction with the MoCA and our clinical assessments. We recommend that future research in Romania prioritize developing and validating a standardized Romanian version of the MMSE to enhance the rigor of cognitive assessments. Furthermore, the lack of Romanian-specific validation data amplifies the known influence of age and educational level on MMSE performance and the potential for floor effects. Cultural or educational factors specific to the Romanian population may further affect the performance of the MMSE in ways not fully understood without dedicated validation research.

Despite these limitations, our study possesses several notable strengths that warrant recognition. Firstly, the prospective design enabled systematic and time-linked data collection of inflammatory markers and cognitive outcomes, reducing recall bias and enhancing the reliability of our findings. Secondly, we employed validated and clinically relevant tools for assessing both delirium (CAM-ICU) and POCD (MMSE and MoCA), ensuring the clinical applicability of our cognitive outcome measures. Thirdly, measuring multiple inflammatory markers (IL-6, CRP, NLR, SII, and SIRI) provides a more comprehensive characterization of the systemic inflammatory response in cardiac surgery patients than studies focusing on fewer markers, offering a richer understanding of inflammatory dynamics. Also, the temporal assessment of inflammatory markers at preoperative, 24 h, and 48 h time points allowed us to capture the dynamic nature of the inflammatory response to the development of POD and POCD, providing valuable insights into the time course of inflammation and cognitive dysfunction. Finally, our study confirms the clinical relevance of well-established perioperative risk factors for POD/POCD in our specific surgical cohort, further validating these factors’ importance and strengthening our findings’ clinical translatability. Furthermore, the homogeneity of our study population, enhanced by excluding patients with high preoperative NLR, allowed for a more precise focus on the acute inflammatory response to cardiac surgery, minimizing the confounding effects of pre-existing chronic inflammation.

## 5. Conclusions

Our study reinforces the multifactorial nature of POCD in cardiac surgery and identifies several key perioperative factors associated with increased risk. Significantly elevated postoperative IL-6 levels emerged as a prominent finding, suggesting a potential role for IL-6 as a marker of inflammatory risk for POD and POCD. Furthermore, our findings highlight the clinical importance of prolonged mechanical ventilation, extended vasopressor support, blood transfusion, renal dysfunction, and elevated postoperative LDH and CK as significant contributors to POD/POCD risk in this cardiac surgery cohort. Advanced age and atrial fibrillation also warrant consideration as important risk factors. These findings highlight the critical need for comprehensive perioperative risk assessment, including consideration of inflammatory status and these identified clinical factors, and for implementing targeted strategies focused on optimizing inflammation control, hemodynamic stability, perfusion, and organ function to effectively mitigate the burden of POD and POCD and improve postoperative cognitive outcomes in cardiac surgery patients.

## Figures and Tables

**Table 1:** Clinical and demographic characteristics of all groups of patients.

Variable	Total (*N* = 88)
Gender (Female), *n* (%)	16 (18.18%)
Age Group, *n* (%)	
≤55 years	21 (18.57%)
55 to 65 years	23 (28.57%)
65 to 75 years	38 (45.71%)
≥75 years	6 (7.15%)
Height, cm	171.06 ± 9.22
Weight, kg	81.35 ± 15.71
BMI, kg/m^2^	27.56 ± 4.47
BMI Group, *n* (%)	
≤18.5 kg/m^2^	0 (0%)
18.5 to 24 kg/m^2^	22 (25%)
≥24 kg/m^2^	66 (75%)
Medical Condition, *n* (%)	
Coronary heart disease, *n* (%)	32 (36.36%)
Diabetes mellitus, *n* (%)	23 (26.13%)
Transfusion	49 (55.68%)
Smokers	19 (21.59%)
POD	17 (19.31%)
POCD	22 (25%)
Surgical approaches, *n* (%)	
Valve surgery	56 (63.63%)
Coronary surgery	32 (36.37%)
CPB time, min	114.95 ± 34.17
Vasopressor support	19.35 ± 9.80
EF%	46.68 ± 5.95
Mean arterial pressure	89.18 ± 9.31

Medical characteristics: BMI (body mass index), POD (postoperative delirium), POCD (postoperative cognitive disfunction), EF% (ejection fraction).

**Table 2:** Comparison of demographic, clinical, surgical, and biomarker variables between patients with and without postoperative delirium (POD) or postoperative cognitive dysfunction (POCD).

Variable	GROUP 1. POD/POCD Group	GROUP 2. No POD/POCD Group	*p*-Value	Cohen’s d	OR	95% Confidence Intervals		Fisher’s Exact Test *p*-Value
Age (average)	66 ± 8.99	60 ± 11.75	0.004	−0.726				
Gender/M	16	55	0.523	0.386	0.386	0.2	2.2	0.53
Weight (average)	78.04 ± 10.75	82.48 ± 16.96	0.244	0.289				
CPB time/min	135.3 ± 43.9	107.65 ± 26.89	*p* < 0.001	−0.916				
MV/hours (average)	27 ± 5.07	14.89 ± 4.40	*p* = 0.004	−0.726				
Diabetes mellitus	7 (31.81%)	16 (24.24%)	0.484		0.377	0.5	4.2	0.57
Postoperative atrial fibrillation	4 (18.18%)	10 (15.15%)	*p* = 0.001		1.618).	0.3	4.4	0.74
NA/hours (average)	26.61 ± 11.80	17.06 ± 7.88	*p* < 0.001	−1.019				
CRP mg/dL at 24 h	78.26 ± 24.46	74.32 ± 21.02	*p* = 0.469					
CRP mg/dL at 48 h	217.28 ± 70.82	177.31 ± 61.556	*p* = 0.020	−0.581				
NLR preop	2.91 ± 1.42	2.64 ± 1.26	*p* = 0.395					
NLR at 24 h	21.4 ± 14.59	15.52 ± 8.75	*p* = 0.038	−0.519				
NLR at 48 h	14.98 ± 8.66	8.81 ± 4.23	*p* = 0.013	−0.623				
IL-6 pg/mL preoperative values	17.04	15.63	*p* = 0.301					
IL-6 pg/mL at 48 h	196.94 ± 131.99	160.79 ± 102.97	*p* = 0.013	−0.623				
IL-17-A pg/mL preoperative values	2.08	4.79	*p* = 0.500					
IL-17-A pg/mL at 48 h	5.16	12.08	*p* = 0.500					
SII preoperative values	490.3 ± 312.83	457.83 ± 229.04	*p* = 0.630					
SII at 24 h	2341.9 ± 1280.82	2309 ± 1521.93	*p* = 0.844					
SIRI preoperative values	1.42 ± 0.97	1.36 ± 1.29	*p* = 0.879					
SIRI at 24 h	12.97 ± 7.93	15.52 ± 9.20	*p* = 0.936					
SIRId (dynamic)	12.49 ± 10.71	12.83 ± 10.42	*p* = 0.959					
Blood transfusion	18 (81%)	30 (45.45%)	*p* = <0.001	2.028	2.028	2.0	28.18	0.001
Smokers	4 (18.18%)	14 (21.21%)	*p* = 0.881		0.088	0.3	3.4	1
EF %(average)	43.2% ± 1.76%	47.72% ± 1.11%	*p* = 0.072					
VEMS 70%	10 (45%)	12(18.18%)	*p* = 0.015					
Creatine kinase IU/L (average)	991.19 ± 876.96	566.07 ± 436.71	*p* = 0.023	−0.570				
Lactate dehydrogenase U/L (average)	429.61 ± 139.14	331.36 ± 98.76	*p* < 0.001	−0.981				
Preoperative creatinine md/dL	1.57 ± 1.11	0.97 ± 0.45	*p* < 0.001	−0.868				
Postoperative creatinine mg/dL	1.95 ± 1.43	1.16 ± 0.53	*p* < 0.001	−0.933				
Glucose/24 h mg/dL	160.09 ± 34.05	153.43 ± 35.18	*p* = 0441					

**Table 3:** Model Summary—Presence of POD/POCD.

Model	Deviance	AIC	BIC	df	ΔΧ^2^	*p*	McFadden R^2^	Nagelkerke R^2^	Tjur R^2^	Cox & Snell R^2^
M_0_	97.805	99.805	102.259	85			0.000		0.000	
M_1_	1.392 × 10^−7^	50.000	111.359	61	97.805	<0.001	1.000	1.000	1.000	0.679

Note. M1 includes age, sex, provenience, weight, CPB time, mechanical ventilation, diabetes mellitus, C-reactive protein (CRP) at 24 h postoperatively, C-reactive protein (CRP) at 48 h postoperatively, preoperative neutrophil-to-lymphocyte ratio, neutrophil-to-lymphocyte ratio (NLR) at 24 h postoperatively, neutrophil-to-lymphocyte ratio (NLR) at 48 h postoperatively, IL-6 at 48 h, IL-17 at 48 h, smokers, preoperative creatinine, postoperative creatinine, lactate dehydrogenase, noradrenaline hours of use, glucose values/24 h, SII preoperative values, SII postoperative values, SIRI preoperative values, SIRI postoperative values.

**Table 4:** Coefficients.

						Wald Test		
Model		Estimate	Standard Error	Odds Ratio	z	Wald Statistic	df	*p*
M_0_	(Intercept)	−1.068	0.247	0.344	−4.321	18.669	1	<0.001
M_1_	(Intercept)	−3607.479	556,892.801	0.000	−0.006	4.196 × 10^−5^	1	0.995
	Age	27.864	4333.550	1.263 × 10^12^	0.006	4.134 × 10^−5^	1	0.995
	Gender (M)	65.211	27,909.801	2.092 × 10^28^	0.002	5.459 × 10^−6^	1	0.998
	Provenience (urban)	−158.730	46,243.628	1.160 × 10^−69^	−0.003	1.178 × 10^−5^	1	0.997
	Weight	3.478	691.307	32.407	0.005	2.532 × 10^−5^	1	0.996
	CPB time/min	6.803	921.187	900.333	0.007	5.454 × 10^−5^	1	0.994
	Mechanical ventilation/hours	23.699	5717.932	1.960 × 10^10^	0.004	1.718 × 10^−5^	1	0.997
	Diabetes mellitus	−7.708	21,123.056	4.493 × 10^−4^	−3.649 × 10^−4^	1.332 × 10^−7^	1	1.000
	C-reactive protein (CRP) at 24 h postoperatively	−3.284	528.525	0.037	−0.006	3.861 × 10^−5^	1	0.995
	C-reactive protein (CRP) at 48 h postoperatively	3.237	408.577	25.457	0.008	6.277 × 10^−5^	1	0.994
	Preoperative neutrophil-to-lymphocyte ratio	−117.037	15,992.441	1.484 × 10^−51^	−0.007	5.356 × 10^−5^	1	0.994
	Neutrophil-to-lymphocyte ratio (NLR) at 24 h postoperatively	7.251	1147.312	1409.735	0.006	3.994 × 10^−5^	1	0.995
	Neutrophil-to-lymphocyte ratio (NLR) at 48 h postoperatively	−5.994	2647.201	0.002	−0.002	5.126 × 10^−6^	1	0.998
	IL-6 at 48 h	1.890	259.199	6.617	0.007	5.315 × 10^−5^	1	0.994
	IL-17 at 48 h	−8.928	1184.888	1.326 × 10^−4^	−0.008	5.678 × 10^−5^	1	0.994
	Smokers	242.676	33,565.572	2.472 × 10^105^	0.007	5.227 × 10^−5^	1	0.994
	Preoperative creatinine	−63.177	151,667.152	3.654 × 10^−28^	−4.165 × 10^−4^	1.735 × 10^−7^	1	1.000
	Postoperative creatinine	122.408	142,604.008	1.450 × 10^53^	8.584 × 10^−4^	7.368 × 10^−7^	1	0.999
	Lactate dehydrogenase	0.295	55.022	1.343	0.005	2.878 × 10^−5^	1	0.996
	Noradrenaline/hours	4.365	1304.360	78.626	0.003	1.120 × 10^−5^	1	0.997
	Glucose/24 h	−2.568	362.526	0.077	−0.007	5.018 × 10^−5^	1	0.994
	Preoperative SII value	−0.648	104.127	0.523	−0.006	3.870 × 10^−5^	1	0.995
	Postoperative SII value	−0.063	10.644	0.939	−0.006	3.537 × 10^−5^	1	0.995
	Preoperative SIRI	112.435	15,629.056	6.760 × 10^48^	0.007	5.175 × 10^−5^	1	0.994
	Postoperative SIRI	10.446	1489.364	34,415.173	0.007	4.919 × 10^−5^	1	0.994

Note. Presence of POD/POCD level “Yes” coded as class 1.

## Data Availability

The original contributions presented in this study are included in the article. Further inquiries can be directed to the corresponding author.
